# Sorting Liquid Droplets by Surface Tension Using Devices with Quasi-Superamphiphobic Coatings

**DOI:** 10.3390/polym12040820

**Published:** 2020-04-04

**Authors:** Yu-Ping Zhang, Di Fan, Xiu-Zhi Bai, Cheng-Xing Cui, Jun Chen, Ren-Long Li, Peng-Fei Liu, Ling-Bo Qu

**Affiliations:** 1 Henan Institute of Science and Technology, Xinxiang 453000, China; ccxcyh@gmail.com (D.F.); amibai@126.com (X.-Z.B.); chengxingcui@hist.edu.cn (C.-X.C.); junchen713@126.com (J.C.); rlli@hist.edu.cn (R.-L.L.); lpf8856@163.com (P.-F.L.); 2 College of Chemistry and Molecular Engineering, Zhengzhou University, Zhengzhou 450001, China; qulingbo@zzu.edu.cn

**Keywords:** surface energy, quasi-superamphiphobic surface, Chinese liquors

## Abstract

Any solid surface with homogenous or varying surface energy can spontaneously show variable wettability to liquid droplets with different or identical surface tensions. Here, we studied a glass slide sprayed with a quasi-superamphiphobic coating consisting of a hexane suspension of perfluorosilane-coated nanoparticles. Four areas on the glass slide with a total length of 7.5 cm were precisely tuned via ultraviolet (UV) irradiation, and droplets with surface tensions of 72.1–33.9 mN m^−1^ were categorized at a tilting angle of 3°. Then, we fabricated a U-shaped device sprayed with the same coating and used it to sort the droplets more finely by rolling them in the guide groove of the device to measure their total rolling time and distance. We found a correlation between ethanol content/surface tension and rolling time/distance, so we used the same device to estimate the alcoholic strength of Chinese liquors and to predict the surface tension of ethanol aqueous solutions.

## 1. Introduction

Inspired by the self-cleaning and water-repellent properties of plants and animals such as lotus leaves [1,2], red rose petals [3], water striders [4,5], and butterflies [6], superhydrophobic and superoleophobic materials have drawn considerable attention in both academic and industrial settings. Hierarchical structures made with low-surface-energy materials can produce surfaces with large static contact angles (SCAs >150°) to water droplets (γ_lv_ = 72.1 mN m^−1^, a representative high-γ_lv_ liquid). Those that exhibit high SCAs (>150°) and low rolling angles (RAs < 10°) to oils (organic liquids) are called superoleophobic surfaces [7]. Surfaces that can repel both water and oils (γ < 30 mN m^−1^) are called superamphiphobic surfaces, which typically have a topography that traps air in pockets [8]. Since organic liquids with lower surface tension can easily spread on many solid substrates, fabricating superamphiphobic surfaces is challenging. Superamphiphobic surfaces are most often produced using chemical etching and plasma treatments [9,10,11,12], though other methods such as electrodeposition [13], spraying [14], and photolithography [15] have been used to coat various substrates such as metals, textiles, and membranes [16,17,18].

Any solid surface with homogeneous or varying surface energy can spontaneously exhibit different wettability toward liquid droplets with different or identical surface tensions (γ). Considering the correlation between wettability and liquid surface tension on solid surfaces, some typical applications include sorting droplets by surface tension as well as estimating the organic concentration and surface tension of organic aqueous solutions [19,20,21]. For example, Kota et al. etched a titanium sheet and fabricated a superomniphobic coating with fluorinated, flower-like TiO_2_ nanostructures [22], tuned the sheet’s surface energy by ultraviolet (UV) irradiation, and used it to categorize droplets by surface tension over a wide range of concentrations (0–60% ethanol aqueous solutions). The droplets with the lowest surface tension for 60% ethanol aqueous solution (28.7 mN m^−1^) ceased at domain 1 due to strong adhesion, and droplets with surface tensions in the range of 37.2–72.1 mN m^−1^ were sorted. Huang et. al recently fabricated a superamphiphobic surface by spraying an ethanol suspension with two sizes of TiO_2_ nanoparticles and perfluorosilane [23]. Their tuned glass with a gradient of solid surface energy or wettability could sort six droplets of ethanol with different concentrations (0–35%) or surface tensions. Moreover, droplets with different concentrations of adenosine triphosphate (ATP) were effectively sorted by the surface after ATP-dependent rolling circle amplification (RCA). Recently, Wang et al. reported a new way to prepare one-way oil-transport fabrics coated with flowerlike ZnO nanorods, fluorinated decyl polyhedral oligomeric silsesquioxanes, and hydrolyzed fluorinated alkylsilane [24]. Changing the irradiation time from 6 h to 30 h broadened the one-way transport of droplets with surface tensions of 22.3–56.7 mN m^−1^. This versatile method estimated the surface tension of a liquid simply by observing its motion on a series of fabrics with different one-way oil-transport selectivity. Later, robust and transparent superhydrophobic surfaces were produced using acid- and base-catalyzed silica particles combined with a candle soot template and chemical vapor deposition (CVD) for chemical modification [25]; three porous silica structures were created that showed different surface tension responsiveness in wettability.

In the current paper, we fabricated a quasi-superamphiphobic coating by spraying a hexane suspension of perfluorosilane-coated nanoparticles onto a glass slide as well as onto a rolling groove with a semi-circular arc. The glass slide was divided into four areas with a total length of ~7.5 mm, and its surface energy and contact angle hysteresis (i.e., the difference between the advancing and receding contact angles) were adjusted in each area by changing the UV irradiation time up to 2 h. The four areas, with identical surface texture but different solid surface energy, allowed high-surface-tension droplets to roll past while trapping low-surface-tension droplets due to adhesion. For comparison, we dropped the same droplets into the guide groove of a semi-circular arc with a diameter of 7 cm, with the same coating, and let these droplets roll back and forth. By measuring their total rolling time or distance, we differentiated the ethanol aqueous solutions with different surface tensions with good reproducibility. In short, we produced a portable device with a super-repellent surface that can sort droplets without tuned areas of solid surface energy. This device is particularly useful for estimating the ethanol volume percentage, namely the alcohol strength (AS), and surface tension of ethanol aqueous solutions such as Chinese liquors in the field. 

## 2. Experimental Section

### 2.1. Preparation of Quasi-Superamphiphobic Surfaces

Glass slides (75 mm long × 25 mm wide × 3 mm thick) were cleaned by sonication in acetone and deionized water thoroughly and dried with nitrogen. Herein, a facile procedure to modify the glass slide by spraying method was attempted. Considering that the wettability of the surface is controlled by the chemical composition and the surface roughness, we added SiO_2_ or TiO_2_ nanoparticles together with one silane coupler of tetraethyl orthosilicate (TEOS) to increase the surface hydrophobicity and roughness. Trichloro(1H,1H,2H,2H-tridecafluoro-n-octyl) silane (FOTS) was added as a coupler for the decrease of low-surface-energy on the surface. The suspension consisted of 1% FOTS, and/or 1% TEOS, and/or 1% SiO_2_ and 3% TiO_2_ nanoparticles in 10 mL hexane mixtures. The uniform suspension was coated on the pretreated glass and U-shaped device carefully by a spray gun (Lotus, Shanghai, China) with a 0.8-mm nozzle diameter by using a nitrogen pressure of 0.3 MPa. Then, the painted glass and U-shaped device were air dried horizontally.

### 2.2. Measurement of Surface Tension, Contact Angles, and Roll Off Angles

Surface tension measurements were carried out at room temperature by the pendent drop method using a contact angle goniometer (TST-200, Shen Zhen Testing Equipment Co.LTD, Shen Zhen, China). Results were expressed as mN/m and are the mean value of triplicate analyses. The contact angles and roll-off angles were measured using the same instrument. The contact angles were measured by advancing or receding 10 ± 0.5 μL droplets on the surface using a micrometer syringe. The roll-off angles were measured by tilting the platform until the 10 ± 0.5 μL droplets rolled off from the surface of the glass slide. At least six measurements were performed on each glass slide.

### 2.3. Tuning Surface Chemistry and Solid Surface Energy via UV Irradiation

Devices with discrete solid surface energy domains were fabricated by UV light irradiating the desired area of the fabricated quasi-superomniphobic surface for the desired time. An XL-1500 UV cross-linker (Spectronics Corp., Westbury, NY, USA), equipped with six 15 W blacklight tubes, was used; one domain of the fabricated glass slides was irradiated at a wavelength of 365 nm while the other domains were masked with a substrate mask. The advancing contact angles measured at different UV irradiation times on fluorinated glass surfaces were used to estimate the solid surface energy by the Fowkes method. 

### 2.4. Characterization of Surface Morphology, Roughness, and Chemical Composition

The surface morphologies of the sprayed glass slides were characterized by scanning electron microscope (SEM, FEI Company, Hillsboro, OR, USA). The surface topography and nanoscale asperities were measured using a three-dimensional (3D) surface profilometer. The roughness of the surfaces was measured using an optical profilometer (GTK-16-0300, Bruker Scientific Company, Waltham, MA, USA). At least three measurements were performed on each surface. The surface element and composition of coating was measured by X-ray photoelectron spectroscopy (XPS, Thermo Fisher, Waltham, MA, USA).

### 2.5. Calculation of the Rolling Distance

The rolling process was videoed and played back by a HUAWEI smart phone (Shenzhen, China) in slow motion mode. Each rolling track was recorded according to the angle mark on the side of the device. The total angles were added according to the marked points (the start point, the top points, and the last end point) that the droplet rolled back and forth along the guide groove.

## 3. Results and Discussion

**Figure 1:**
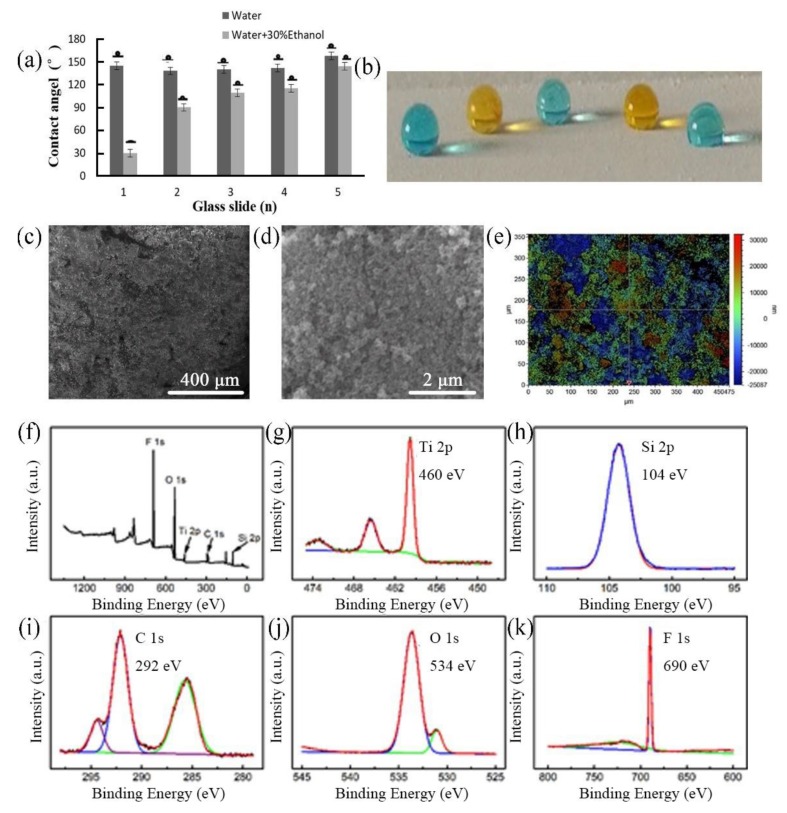
(**a**) Contact angles (CAs) of water and 30% ethanol on glass surfaces treated with various coatings: (1) 1% trichloro(1H,1H,2H,2H-tridecafluoro-n-octyl) silane (FOTS) + 1% nano SiO_2_; (2) 1% FOTS + 1% tetraethyl orthosilicate (TEOS) + 3% TiO_2_; (3) 1% FOTS + 1% nano SiO_2_ + 3% TiO_2_; (4) 1% FOTS + 1% nano SiO_2_ + 1% TEOS; (5) 1% FOTS + 1% nano SiO_2_ + 1% TEOS + 3% TiO_2_. (**b**) Images of the droplets of ethanol aqueous solutions on the quasi-superamphiphobic surface of Slide No. 5. Droplets (from left to right) of water, water + 10% ethanol, water + 20% ethanol, water + 30% ethanol, and water + 40% ethanol (γ*_lv_* = 33.9 mN m^−1^), showing gradually decreasing apparent contact angles on the quasi-superamphiphobic surface. (**c**–**d**) Scanning electron microscopy images showing the morphology of the optimal glass slide, Slide No. 5. (**e**) Roughness mapping with a *R*_rms_ of 9.1 ± 0.1 μm. (**f**) X-ray photoelectron spectroscopy (XPS) survey spectra of Slide No. 5. (**g**–**k**) XPS core level spectra of Ti 2p, Si 2p, C 1s, O 1s, and F 1s peaks.

A mixture of a 10 mL hexane suspension was prepared and mixed fully by a vortex oscillator for 30 min, containing 1% (v/v) trichloro(1H,1H,2H,2H-tridecafluoro-n-octyl)silane (FOTS), 1% (v/v) tetraethyl orthosilicate (TEOS), 1% (*w*/*v*) SiO_2_ (20–40 nm), and/or 3% (*w*/*v*) TiO_2_ nanoparticles (~21 nm). To prepare the super-repellant surface, we optimized various suspension solutions (Nos. 1–5) by step-by-step addition of active FOTS, TEOS, SiO_2_, and TiO_2_ nanoparticles in different amounts. contact angles (CAs) in the range of 138–152° appeared for the deionized water (γ_lv_ = 72.1 mN m^−1^) and 30–144° for the 30% ethanol aqueous solution (γ_lv_ =37.2 mN m^−1^) (see Figure 1). The glass slide sprayed with the No. 5 suspension solution showed optimal liquid repellence, with rolling angles of 1.3° and 2.9° for water and the 30% ethanol solution, respectively. This coating still repelled water even after being immersed in oil (hexane), and it showed water jetting and water droplets bouncing on the quasi-superhydrophobic surface with no residual adhesion on the glass slide (see Video S1). On this surface, both water and 30% ethanol were primarily in the Cassie–Baxter state, which agrees with our previous study on laboratory filter paper [26].

**Figure 2:**
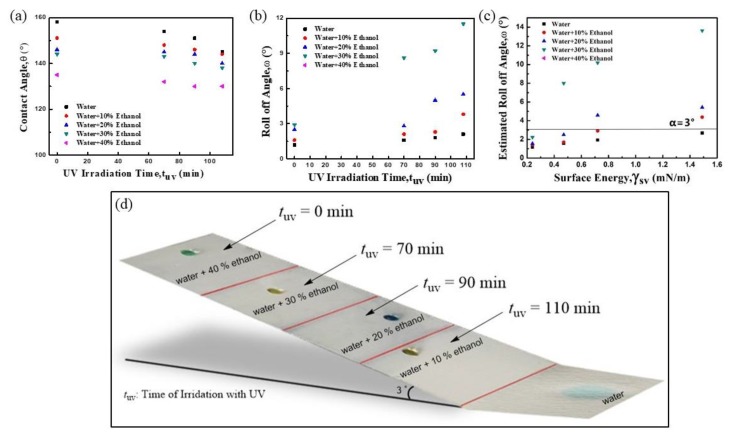
(**a**) Apparent contact angles of droplets on Slide No. 5, decreasing with increasing ultraviolet (UV) irradiation time. (**b**) Measured roll-off angles of 10 μL ethanol solution droplets on the slide increased faster than those of 10 μL water droplets with increasing UV irradiation time. (**c**) Estimated roll-off angles of various 10 μL droplets on the slide with different solid surface energies. (**d**) Schematic showing the sorting of 10 μL droplets using the slide tilted at 3° with four areas of different surface energy.

As shown by scanning electron microscopy, our quasi-superamphiphobic coating exhibited nano/micro structures, produced from the adherence between FOTS/TEOS and both nanoparticles with different sizes (see Figure 2a,b). Slide No. 5 had an average roughness (*R*_a_) of 9.1 ± 0.1 μm (Figure 2c). Elemental analysis found Ti 2p, Si 2p, C 1s, O 1s, and F 1s on the surface of the glass slide (Figure 2d). The Ti 2p high-resolution spectrum was deconvoluted into peaks at 460 eV (Ti 2p 1/2) and 458.6 eV (Ti 2p 3/2), indicating TiO_2_. The Si 2p peak appeared at a binding energy of 104 eV, referring to Si, and the C 1s profile was characterized by two typical peaks at 287 and 292 eV. In addition, the O 1s profile was characterized by two typical peaks at 534 and 531 eV (see Figure 2d). The elemental atomic contents were 1.75% Ti 2p, 15.8% Si 2p, 18.5% C 1s, 31.7% O 1s, and 32.3% F 1s. 

We tailored the resulting quasi-superamphiphobic glass slide by shining UV light on four areas. The photocatalytic activity of TiO_2_ in the coating allowed us to precisely tune the solid surface energy. After UV irradiation, the TiO_2_ nanoparticles generated holes that readily reacted with lattice oxygen, creating surface oxygen vacancies. These vacancies coordinated to water molecules in air, which increased the surface hydrophilicity [27,28]. Here, areas 2–4 were irradiated for 70, 90, and 110 min, respectively, while area 1 was not irradiated. With increasing UV irradiation time, the solid surface energy increased from area 1 (0.24 mN m^−1^) to area 4 (1.49 mN m^−1^), determined by the Fowkes method (Figure S1) [29,30]. Likewise, with increasing UV irradiation time, the apparent contact angles gradually decreased and the contact angle hysteresis increased for both water and the four ethanol aqueous solutions (Figure 2a,b). The measured roll-off angles of the 10 μL ethanol solution droplets with less surface tension on the glass slide increased faster than those of the 10 μL water droplets.

The tilted glass slide sorted droplets based on a balance between the work done by gravity and the work expended by adhesion, as described in Equation (1) [22,23]:

*Vρ*gsin ω ≈ γ*_lv_D*_TCL_ (cosθ^*^_rec_ − cosθ^*^_adv_) (1)

where *V* is the volume of the droplet, *ρ* is the liquid density, g is the acceleration of gravity, and ω is the roll-off angle of the droplet. *D*_TCL_ is the width of the solid–liquid–vapor contact line perpendicular to the rolling direction, θ*_r__ec_ and θ*_adv_ are the apparent receding and advancing contact angles, and γ*_lv_* is the surface tension of the liquid on the solid surface. Thus, droplets with certain higher surface tension should roll-off the solid surface with a certain surface energy γ_sv_ and appropriate inclination angle α (here, ω < α), while droplets with lower surface tension and ω > α will be trapped on the corresponding areas. 

**Figure 3:**
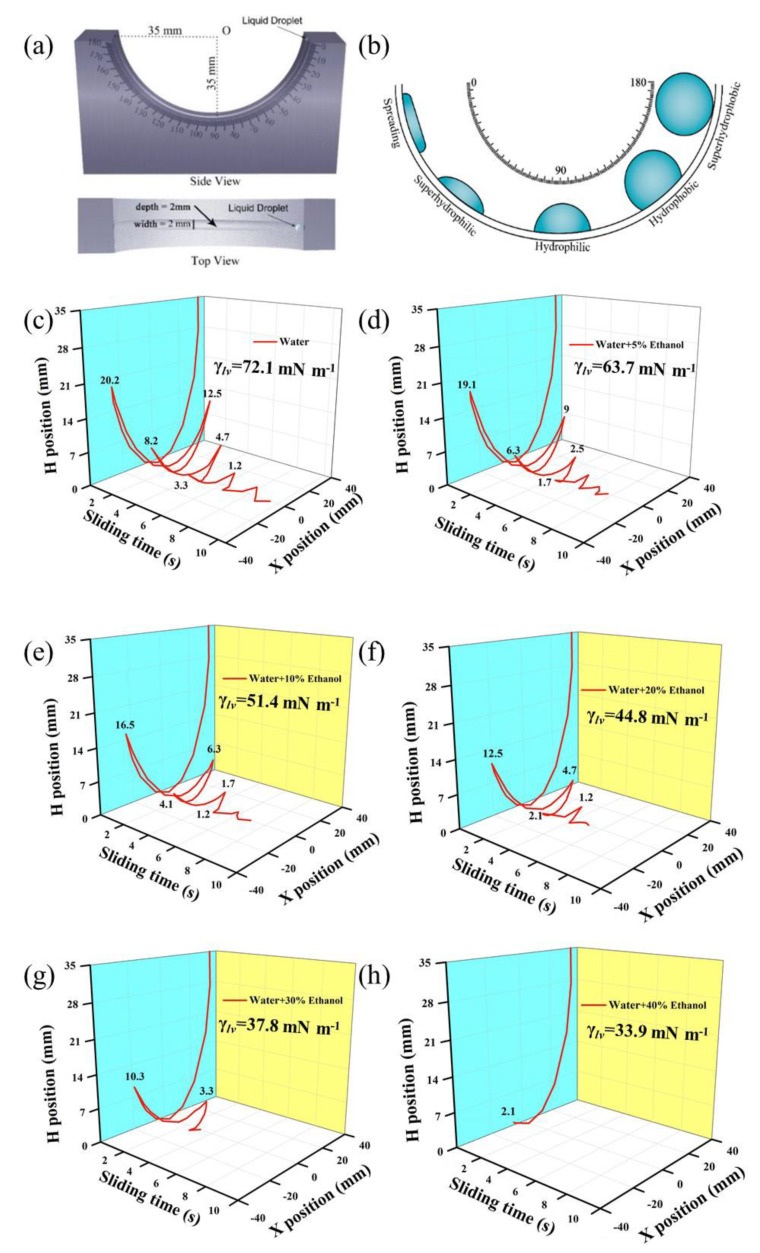
(**a**) Side and top views of our device for droplet sorting, surface tension estimation, and AS prediction. (**b**) Images of droplets with various surface tensions in the guide groove. (**c**–**h**) Schematics showing the fine sorting of droplets by surface tension using our device and measuring their rolling time or distance.

The surface tensions of various ethanol aqueous solutions (0–40%) were determined by the pendant drop method [31,32,33] and selected for droplet sorting. We estimated the roll-off angles for five 10 µL droplets in each of the four areas by measuring their θ*_rec_ and θ*_adv_ (Table S1). Based on the estimated roll-off angles (Figure 2c), when Slide No. 5 is set at an angle α = 3°, droplets of the 40% ethanol aqueous solution (γ*_lv_* = 33.9 mN m^−1^) should get trapped in area 1; droplets of 30% ethanol (γ*_lv_* = 37.8 mN m^−1^) should roll past area 1 but get trapped in area 2; droplets of water + 20% ethanol (γ*_lv_* = 44.8 mN m^−1^) should roll past areas 1 and 2 but get trapped in area 3; droplets of water + 10% ethanol (γ*_lv_* = 51.4 mN m^−1^) should roll past areas 1, 2, and 3 but get trapped in area 4; and droplets of water (γ*_lv_* = 72.1 mN m^−1^) should roll past all areas. These predictions matched reasonably well with our experimental results shown in Figure 3c,d. The whole process to sort five droplets using the tuned glass slide at a tilt angle of 3° (based on the estimated roll-off angles) is shown in Figure 3d (also see Video S2). Five droplets, with surface tensions of 33.9–72.1 mN m^−1^, were sorted on the four areas but with limited resolution and discrimination. That is, the droplets with similar surface tensions were difficult to sort without tuning the solid surface energy further. For example, the droplets of the 5% ethanol aqueous solution and the 10% ethanol solution rolled off and were both trapped in area 4, showing less discrimination and reproducibility [22,23].

Thus, we improved the discrimination degree by using a U-shaped analytical device treated with identical coating, sorting droplets with surface tensions of 33.9–72.1 mN/m according to their motion as they rolled back and forth in the guide groove (Figure 3a).

By recording the entire motion of a droplet along the guide groove in slow motion with a smartphone, we calculated its total rolling time and distance. In calculating the total distance, by marking the highest position each time the droplet moved back and forth in the groove, the total rolling or sliding angle (A1 + A2 + A3 + … + An) could be added together to calculate the total rolling distance (D, mm) as

(2) $$D=\frac{\left(A1+A2+\cdots \mathrm{An}\right)\times 2\pi \times R}{360^{{}^{\circ}}}$$

where A1, A2…An are the rolling angles along the circular arc for the first, second, and *n* time, respectively, and R is the radius of the semi-circular metal platform.

Water droplets of various volumes (5–50 μL) were dropped in the guide groove, and the total rolling time and distance was recorded in ranges of 6–23 s and 209–701 mm, respectively. Further increasing the droplet volume caused the droplet to roll out of the guide groove unexpectedly. The droplet volume correlated well to the rolling time and distance, with correlation coefficients (R) of 0.9980 and 0.9897, respectively (Figures S2 and S3). Thus, the droplets could be sorted over a wider range of surface tensions by increasing the droplet volume. 

Generally, 10 μL droplets of various ethanol aqueous solutions (0–40%) were carefully dispensed into the guide groove from the top of the U-shaped analytical device using a syringe capped with a hydrophobic needle. The droplet slides along the guide groove from the top, rolls across the lowest point in the groove, and rolls up to the first highest point along the groove. After rolling back and forth several times, the droplet finally stops somewhere in the groove, most often at the bottom. Droplets with less surface tension slide for less time and distance due to stronger adhesion. The change of the droplet potential energy remains as the sum of the energy dissipation and kinetic energy change during the droplet rolling back and forth in the guide groove. Therefore, it satisfies the equation ΔE_total_ = ΔE_loss_ + ΔE_kinetic_, where ΔE_total_ (ΔE_total_ = mgΔh, where m is the mass of the droplet, g is the gravitational acceleration, and Δh is the elevation between the droplet location and the lowest point in the guide groove) represents the potential energy change of the droplet along the arc hydrophobic surface. ΔE_loss_ is the dissipation energy, which is created by deformation force of the droplet, adhesion force, shear force, and air-drag force during the movement of the liquid droplet [34]. For the liquid droplets with larger surface tension, it is easily understood that it can move easily on the superhydrophobic surface with less ΔE_loss_; thus, larger ΔE_kinetic_ will drive the liquid droplet to move a longer time or distance along the groove [20,35].

Droplets rolled from the top (about 35 mm above the bottom of the groove), rolled across the bottom, then rolled up to the first highest point (20.2 mm above the bottom). The water droplet that rolled for the longest time and distance rolled back and forth about 10 times with a distance of 359 mm over 10 s. As the ethanol concentration gradually increased to 40%, the rolling time and distance decreased to 1.5 s and 67 mm, respectively (see Video S3). 

Using this simple operation, we sorted the six droplets by surface tensions of 33.9–72.1 mN m^−1^. Compared with our tuned glass slide and previous reports with a rolling length of 60–75 mm [22,23], we obtained higher resolution and discrimination, with rolling distances in the range of 67–359 mm. That is, we sorted the droplets with closer surface tensions and over a wider range of surface tensions using our device. More importantly, we sorted the droplets by rolling time in a range of 1.5–10.1 s by simply recording them in slow motion (1/4 normal speed) with a smartphone. 

The rolling motions of the droplets are illustrated in the simulated 3D diagram shown in Figure 3. Six droplets with different surface tensions were differentiated by rolling time, rolling distance, number of rolling cycles, and the first highest position. As the surface tension gradually decreased from 72.1 mN m^−1^ to 33.9 mN m^−1^, the first highest position gradually decreased from 20.2 mm to 2.1 mm. Each droplet was rolled eight times, and the relative standard derivation (RSD) of the total rolling distance was less than 1% (Table S2). These results show that the sprayed coating was not damaged after rolling droplets with surface tensions of 33.9–72.1 mN m^−1^ many times, and this stable surface could be used for rolling over 100 times without an apparent decrease in reproducibility. However, the coating gradually was deteriorated by infiltration of a droplet with a surface tension lower than 30 mN m^−1^, because the stronger adhesion of this droplet decreased the reproducibility of the rolling time and distance. 

Thus, the relationship between the rolling time/distance and ethanol volume content was computed. Chinese liquors mostly consist of ethanol and water with trace flavor additives, so they can be regarded as typical ethanol–water mixtures, with their alcohol strength (AS) expressed in an ethanol volume percent by total volume, estimated according to their rolling time or distance. Using a standard regression curve, we estimated the AS and surface tension of some samples of real Chinese liquors. Here, Chinese liquors of Jinjiu (labeled AS of 35%) and Maotai Wangzhi (labeled AS of 52%) were diluted to various concentrations. We tested three diluted Jinjiu samples with AS values of 20%, 30%, and 35% as well as three diluted Maotai samples with AS values of 10.6%, 21.2%, and 31.8%, respectively.

After determining or calculating the rolling time and distance for 10 μL droplets of these liquors, the estimated AS results agreed well with the labeled AS values, and we could predict about 80% the AS values of these samples based only on rolling time or distance (see Table S3). The correlation between alcohol strength (AS) and *T*_total_ was obtained as = 0.1383 *T*^2^ − 6.1804 *T* + 49.894, with a regression coefficient (*R*) of 0.9836 (see Figure S4). In addition, the correlation between AS and D_total_ was obtained as AS = 0.0063 *D*^2^ − 1.7306*D* + 52.569, with a regression coefficient (*R*) of 0.9880 (see Figure S5). 

Simulated tracks of the real liquor samples are also illustrated (see Figure S6), and the diluted liquors were recorded while rolling to compare them with standard solutions of similar ethanol volume contents (see Video S4). Similar rolling times and cycle numbers appeared, indicating the potential of predicting the relative AS values. Moreover, the surface tensions (γ_lv_) of the selected six liquors were calculated using regression equations based on rolling time and distance (see Figures S7 and S8). Their calculated γ_lv_ values agreed well with the experimental values, with a maximum relative error of ~10% (see Table S4). Thus, our method is a simple, fast way to estimate the AS and surface tensions of Chinese liquors using a portable device, which is useful in the field where lab platforms and analytical instruments are not available. 

## 4. Conclusions

We produced U-shaped devices sprayed with a quasi-superamphiphobic coating, which we used to sort droplets with different surface tensions. Using it, we determined the surface tension and estimated the alcohol strength of Chinese liquors and showed it could be used over a hundred times to sort droplets by surface tension in the range of 33.9–72.1 mN m^−1^. Compared with tuned glass slides, our device was simpler and faster, and it did not require lengthy tuning of solid surface energy or an expensive high-speed camera. Based on our methodology and mechanism, higher resolution may be achieved, and wider range of surface tensions are possibly determined by increasing the droplet volume or changing the semi-circular arc diameter of the device. Along with our ongoing studies of the relative mechanism of energy loss of a rolling drop, we believe this method of sorting droplets and determining surface tension will lead to inexpensive, energy-efficient analytical devices for chemical assays in the field.

## Supplementary Materials

The following are available online at https://www.mdpi.com/2073-4360/12/4/820/s1, Table S1: Apparent advancing and apparent receding contact angles, and the estimated roll-off angles of different water–ethanol mixtures on each of the discrete domains shown in Figure 2a–c of the main manuscript, Table S2: Total rolling distance (cm) and relative standard deviation (RSD, %) using the guide groove for ethanol aqueous droplets in the concentration range of 0–40%, Table S3: The comparative results and relative errors (REs) between the labeled AS by suppliers and the estimated AS for 6 commercial liquor samples using the total rolling time (RT) and rolling distance (RD), Table S4: Comparative results of surface tension for the diluted liquors between the determined value by instrument and the predicted value by the fitting quadratic equation of total rolling time (RT) and rolling distance (RD), Figure S1: The wettability transformation under UV irradiation, Figure S2: The relationship between the rolling time and water droplet volume, Figure S3: The relationship between the total rolling distance and water droplet volume, Figure S4: The standard curve between AS and rolling time used to predict the ethanol volume content or AS for real liquors, Figure S5: The standard curve between AS and rolling distance used to predict the ethanol volume content or AS for real liquors, Figure S6: Track of diluted liquor droplets rolling back and forth in the U-shaped device used to estimate the ethanol volume content (or AS values) and surface tension of Chinese liquors, Figure S7: The standard curve between rolling time and surface tension used to predict the surface tension values of real liquors. The real values of surface tension for ethanol aqueous solutions were determined by the pendent drop method. Three Jinjiu liquors with the labeled AS values of 20%, 30%, and 35% were selected for the γlv estimation, as well as the diluted Maotai liquors with the AS values of 10.6%, 21.2%, and 31.8%, respectively, Figure S8: The standard curve between rolling distance and surface tension used to predict the surface tension values of real liquors. The real values of surface tension for ethanol aqueous solutions were determined by the pendent drop method. Three Jinjiu liquors with the labeled AS values of 20%, 30%, and 35% were selected for the γlv estimation, as well as the diluted Maotai liquors with the AS values of 10.6%, 21.2%, and 31.8%, respectively, Video S1: Water jetting on the quasi-superhydrophobic surface (AVI), Video S2: Droplet sorting using the tuned glass slide (AVI), Video S3: Standard rolling curve used to test the alcohol strength (AS) (AVI), Video S4: AS estimation of real samples (AVI).
