# Supplementary material for: Sorting Liquid Droplets by Surface Tension Using Devices with Quasi-Superamphiphobic Coatings
Source: Polymers (Basel). 2020 Apr 4;12(4):820. doi: 10.3390/polym12040820 (PMC7240524; doi:10.3390/polym12040820)
Supplement: Supplementary file 1 [file polymers-12-00820-s001.zip › polymers-757548-supplementary for final/polymers-757548-supplementary for final.docx]

Sorting Liquid Droplets by Surface Tension Using Devices with Quasi-Superamphiphobic Coatings

Yu-Ping Zhang, Di Fan, Xiu-Zhi Bai, Cheng-Xing Cui, Jun Chen, Ren-Long Li,
Peng-Fei Liu and Ling-Bo Qu

In general, water droplets can easily roll off from a superhydrophobic surface due to the high apparent contact angle *θ** larger than 150° and low contact angle hysteresis Δθ* lower than 10°. Usually, the superhydrophobic surface is fully or partly infiltrated and deteriorated by liquid droplets with lower surface tension. When the liquid droplets roll off from a tilted surface plate with a fixed angle, a balance between work done due to gravity and work expanded because of adhesion should be reached, as shown in the following equation:

| *Vρ*g sin*ω ≈ γ_lv_ D_TCL_* (cos*θ*_rec_−*cos *θ*_adv_*) | (S1) |
| --- | --- |

where *V* is the volume of the liquid droplet, *ρ* is the density of the liquid, g is the acceleration of gravity, and ω is the roll-off angle of the droplet (the minimum angle the surface must be tilted for droplet to roll off). *D_TCL_* is the width of the solid–liquid–vapor contact line perpendicular to the rolling direction, *θ*_rec_* and *θ*_adv_* are the apparent receding and advancing contact angles, and *γ_lv_* represents the interaction between liquid and solid surface. In addition, when the shape of the droplet does not deviate significantly from a spherical cap, the width of *D_TCL_* can be calculated as follows:

| $D_{TCL}=2\cos\left( \bar{\theta}^{*}-\frac{\pi}{2} \right)\left[ \frac{3V}{\pi\left( 2-3\cos\bar{\theta}^{*}+\cos^{3} \bar{\theta}^{*} \right)} \right]^{\frac{1}{3}}$ | (S2) |
| --- | --- |

Here, $\bar{\theta}$*** is the average apparent contact angle, given as:

| $\cos\bar{\theta}^{*}=\frac{\cos\theta_{adv}^{*}+\cos\theta_{rev}^{*}}{2}$ | (S3) |
| --- | --- |

In the case of droplets with fixed volume, but different surface tension (or different interaction between liquid and solid surface), droplets with lower *γ_lv_* (or stronger interaction with solid surface) adhere more to a superhydrophobic surface which results in a higher *D*_TCL_ and higher *Δθ*,* and then displays higher ω. A water droplet with a *γ_lv_* of 72.1 mN m^-1^ will freely roll off the titled surface and display a lower ω. Thus, it could be predicted that liquid droplets with certain higher surface tension (or weaker interaction with solid surface) will roll off from the solid surface with certain surface energy and appropriate tilt angle α (here ω < *α*), while droplets with lower surface tension and ω > *α* will adhere to the surface. For our selected liquid droplets with a *γ_lv_* in the range of 33.9–72.1 mN m^-1^, their α values are nearly close to 90° when they are removed and dropped at the top of the guide groove. Based on the results of *θ*_rec_*, *θ*_adv_* and ω determined on the quasi-superamphiphobic surface, we estimate the RAs for ethanol aqueous solutions on the glass slide. The estimated roll-off angles of different liquids in Figure 2c of the main manuscript are calculated using Equations (S1)–(S3) (see Table S1).

All liquid droplets can roll off from the starting point along the quasi-superamphiphobic groove. When the rolling droplet reaches one position along the groove, the *α* value gradually decreases to zero at the bottom of groove. The droplet will keep moving back and forth due to its force of inertia until it stops by air resistance and adhesion work expended. The different adhesion work expanded results in different rolling distance and time for droplets with different surface tensions. In this way, we can easily sort the liquid droplets with different surface tensions without lengthy tuning of the solid surface energy of the guide groove.

**Table S1.** Apparent advancing and apparent receding contact angles, and the estimated roll-off angles of different water–ethanol mixtures in each of the discrete domains shown in Figure 3a–c of the main manuscript.

| Item | Angle(º) | Water | Water +10% Ethanol | Water +20% Ethanol | Water +30% Ethanol | Water +40% Ethanol | Surface Energy (γ*_sv_*, mN m^−1^) |
| --- | --- | --- | --- | --- | --- | --- | --- |
| Surface Tension  (mN m^−1^) | - | γ*_lv_* = 72.1 | γ*_lv_* = 51.4 | γ*_lv_* = 44.8 | γ*_lv_* = 37.8 | γ*_lv_* = 33.9 | - |
| Domain 1 | *θ*^*^_adv_ | 158 | 151 | 146 | 144 | 135 | (*t_UV_* = 0 min; γ*_sv_* = 0.24 mN m^−1^) |
|  | *θ*^*^_rec_ | 156 | 149 | 143 | 137 | No sliding |  |
|  | *ω* | 1.2 | 1.4 | 1.6 | 2.2 |  |  |
| Domain 2 | *θ**_adv_ | 154 | 148 | 145 | 143 | 132 | (*t_UV_* = 70 min; γ*_sv_* = 0.47 mN m^−1^) |
|  | *θ**_rec_ | 152 | 145 | 141 | 134 | No sliding |  |
|  | *ω* | 1.6 | 1.7 | 2.5 | 8.0 |  |  |
| Domain 3 | *θ**_adv_ | 151 | 146 | 144 | 140 | 130 | (*t_UV_* = 90 min; γ*_sv_* = 0.72 mN m^−1^) |
|  | *θ**_rec_ | 149 | 143 | 139 | 130 | No sliding |  |
|  | ω | 1.9 | 2.9 | 4.6 | 10.2 |  |  |
| Domain 4 | *θ**_adv_ | 145 | 144 | 140 | 138 | 130 | (*t_UV_* = 110 min; γ*_sv_* = 1.49 mN m^−1^) |
|  | *θ**_rec_ | 142 | 140 | 135 | 126 | No sliding |  |

**Table S2.** Total rolling distance (cm) and relative standard deviation (RSD, %) using the guide groove for ethanol aqueous droplets in the concentration range of 0–40%.

| Liquid  Droplet | γ_lv_  (mN m^-1^) | Total Rolling Distance (cm) | | | | | | | | D_average_ (cm) | RSD (%) |
| --- | --- | --- | --- | --- | --- | --- | --- | --- | --- | --- | --- |
|  |  | run 1 | run 2 | run 3 | run 4 | run 5 | run 6 | run 7 | run 8 |  |  |
| water | 72.1 | 35.9 | 35.8 | 36.7 | 35.7 | 35.2 | 36.2 | 35.5 | 36.1 | 35.9 | 0.4 |
| 5% | 63.7 | 27.1 | 26.7 | 26.9 | 26.3 | 26.1 | 26.0 | 26.2 | 27.3 | 26.6 | 0.5 |
| 10% | 51.4 | 25.1 | 24.6 | 24.9 | 25.1 | 25.1 | 25.0 | 24.3 | 24.5 | 24.8 | 0.3 |
| 20% | 44.8 | 22.0 | 21.2 | 20.8 | 21.0 | 22.4 | 21.6 | 21.0 | 21.7 | 21.5 | 0.5 |
| 30% | 37.8 | 15.9 | 15.5 | 15.9 | 15.2 | 15.3 | 15.9 | 15.6 | 15.3 | 15.6 | 0.3 |
| 40% | 33.9 | 6.7 | 6.7 | 6.9 | 6.7 | 6.6 | 6.7 | 6.6 | 6.7 | 6.7 | 0.1 |

**Table S3.** The comparative results and relative errors (REs) between the labeled alcohol strength (AS) by suppliers and the estimated AS for 6 commercial liquor samples using the total rolling time (RT) and rolling distance (RD).

| Chinese Liquor | AS Labeled (%vol) | AS Estimated by RT (%vol) | RE (%) | AS Estimated by RD (%vol) | RE (%) |
| --- | --- | --- | --- | --- | --- |
| Jingjiu | 20 | 23.8 | 19 | 22.4 | 12 |
|  | 30 | 31.3 | 4.3 | 30.5 | 1.5 |
|  | 35 | 35.9 | 2.5 | 33.1 | 5.4 |
| Maotai wangzhi | 10.6 | 13.2 | 24.5 | 12.8 | 20.7 |
|  | 21.2 | 26.1 | 23.1 | 23.3 | 9.9 |
|  | 31.8 | 33.2 | 4.4 | 31.2 | 1.8 |

**Table S4.** Comparative results of surface tension for the diluted liquors between the determined value by instrument and the predicted value by the fitting quadratic equation of total rolling time (RT) and rolling distance (RD).

| Chinese Liquor | AS Labeled (%vol) | γ_Det._  (mN m^−1^) | γ_pred._  by RT  （mN m^−1^） | RE (%) | γ_pred._ by RD  （mN m^−1^） | RE (%) |
| --- | --- | --- | --- | --- | --- | --- |
| Jingjiu | 20 | 41.8 | 40.8 | 2.3 | 42.4 | 1.4 |
|  | 30 | 37.5 | 36.7 | 2 | 37.1 | 1.1 |
|  | 35 | 35.3 | 35.0 | 0.6 | 35.8 | 1.5 |
| Maotai wangzhi | 10.6 | 51.6 | 49.6 | 4 | 51.4 | 0.4 |
|  | 21.2 | 42.5 | 39.1 | 7.9 | 41.7 | 1.8 |
|  | 31.8 | 38.6 | 35.9 | 6.9 | 36.7 | 5.1 |


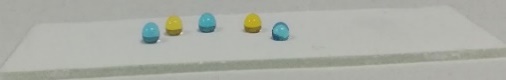

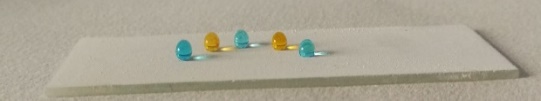


**Figure S1.** The wettability transformation under UV irradiation.

The inlet images stand for the droplets’ wetting states on domain 1 and domain 4, respectively. The droplets (from left to right: water, 10%, 20%, 30%, 40% ethanol solution) were dyed with methylene blue and methyl orange, respectively.

**Figure S2.** The relationship between the rolling time and water droplet volume.

**Figure S3.** The relationship between the total rolling distance and water droplet volume.

In order to predict the AS values of real samples, six ethanol aqueous solutions were initially selected in the range of 0–40% for the construction of the standard curve. The quadratic equation model fitted could be used for the estimation of AS values of real samples, based on total rolling time or distance in Figures S4 and S5. The AS values estimated by the equation were close to the actual values labeled by suppliers. These dots of six real liquors scattered closely to the standard curve. The updated model explained about 80% of the ethanol content variation based on rolling time or distance of these labeled liquors (see Table S3). It provided an additional method for the simple and rapid estimation of the AS of Chinese liquors.

**Figure S4.** The standard curve between AS and rolling time used to predict the ethanol volume content or AS for real liquors.

Three Jinjiu liquors with the labeled AS values of 20%, 30%, and 35% were selected for the AS estimation, as well as three diluted Maotai liquors with the AS values of 10.6%, 21.2%, and 31.8%, respectively.

**Figure S5.** The standard curve between AS and rolling distance used to predict the ethanol volume content or AS for real liquors.

Three samples of Jinjiu liquors with the labeled AS values of 20%, 30%, and 35% were selected for the AS estimation, as well as three diluted Maotai liquors with the AS values of 10.6%, 21.2%, and 31.8%, respectively.

**Figure S6.** Track of diluted liquor droplets rolling back and forth in the U-shaped device used to estimate the ethanol volume content (or AS values) and surface tension of Chinese liquors.

In order to test the predictive model of surface tension, six solutions were selected in the range of 0%–40% ethanol for the construction of a standard curve. The quadratic equation model fitted could be used for the estimation of surface tension, based on total rolling time or distance in Figures S7 and S8. These dots of six real liquors scattered closely to the standard curve. The updated model explained over 90% of the variation of surface tension based on rolling time or distance for the selected six liquors (see Table S4). It provided an additional method for the simple and rapid estimation of surface tension for Chinese liquors.

**Figure S7.** The standard curve between rolling time and surface tension used to predict the surface tension values of real liquors. The real values of surface tension for ethanol aqueous solutions were determined by the pendent drop method. Three Jinjiu liquors with the labeled AS values of 20%, 30%, and 35% were selected for the γ_lv_ estimation, as well as the diluted Maotai liquors with the AS values of 10.6%, 21.2%, and 31.8%, respectively.

**Figure S8.** The standard curve between rolling distance and surface tension used to predict the surface tension values of real liquors. The real values of surface tension for ethanol aqueous solutions were determined by the pendent drop method. Three Jinjiu liquors with the labeled AS values of 20%, 30%, and 35% were selected for the γ_lv_ estimation, as well as the diluted Maotai liquors with the AS values of 10.6%, 21.2%, and 31.8%, respectively.
